# Stakeholder Feedback of Electronic Medication Adherence Products: Qualitative Analysis

**DOI:** 10.2196/18074

**Published:** 2020-12-01

**Authors:** Sadaf Faisal, Jessica Ivo, Aidan McDougall, Tejal Patel

**Affiliations:** 1 School of Pharmacy University of Waterloo Kitchener, ON Canada; 2 Centre for Family Medicine Family Health Team Kitchener, ON Canada; 3 Schlegel-University of Waterloo Research Institute of Aging University of Waterloo Waterloo, ON Canada

**Keywords:** medication nonadherence, technology, aged, patient preferences, eHealth, qualitative research, adherence

## Abstract

**Background:**

Medication management among older adults continues to be a challenge, and innovative electronic medication adherence products have been developed to address this need.

**Objective:**

The aim of this study is to examine user experience with electronic medication adherence products, with particular emphasis on features, usefulness, and preferences.

**Methods:**

Older adults, caregivers, and health care providers tested the usability of 22 electronic medication adherence products. After testing 5 products, participants were invited to participate in a one-on-one interview to investigate their perceptions and experiences with the features, usefulness, and preference for electronic medication adherence products tested. The interviews were audio recorded, transcribed, and analyzed using exploratory inductive coding to generate themes. The first 13 interviews were independently coded by 2 researchers. The percentage agreement and Cohen kappa after analyzing those interviews were 79% and 0.79, respectively. A single researcher analyzed the remaining interviews.

**Results:**

Of the 37 participants, 21 (57%) were older adults, 5 (14%) were caregivers, and 11 (30%) were health care providers. The themes and subthemes generated from the qualitative analysis included product factors (subthemes: simplicity and product features, including availability and usability of alarms, portability, restricted access to medications, and storage capacity) and user factors (subthemes: sentiment, affordability, physical and cognitive capability, and technology literacy and learnability).

**Conclusions:**

Electronic medication adherence products have the potential to enable independent medication management in older adults. The choice of a particular product should be made after considering individual preferences for product features, affordability, and the sentiment of the users. Older adults, caregivers, and health care providers prefer electronic medication adherence products that are simple to set up and use, are portable, have easy-to-access medication compartments, are secure, and have adequate storage capacity.

## Introduction

### Background

The older adult population is increasing rapidly worldwide, with a projection of approximately 1.5 billion individuals aged 65 years or older by 2050 [1]. In North America and Europe alone, individuals aged 65 years or older will account for 19.1% of the total population by 2050 [1]. In 2016, a Canadian study showed that 65.7% of adults aged 65 years or older were prescribed 5 or more medications, 26.5% were prescribed 10 or more medications, and 8.4% were prescribed 15 or more medications [2]. Approximately 50% of older adults are nonadherent to their medications [3]. Poor medication adherence leads to nonoptimal management of diseases; increased emergency room visits; hospitalization; and, ultimately, poor quality of life [4].

The incidence of nonadherence is highly prevalent in the geriatric population because of multiple factors [5]. As people age, they are diagnosed with multiple chronic health conditions that often require complex medication regimens, with multiple medications, various dosage forms, and complicated medication schedules [6]. In addition, older adults face significant issues with medication management because of impaired functional capabilities, such as poor vision, hearing loss, dexterity issues, or cognitive capabilities [6-8]. These functional and cognitive capabilities are imperative for managing complex medication regimens; therefore, older adults with such deficits are at higher risk of medication mismanagement [9].

Numerous medication management strategies are being used by older adults to improve adherence, such as pillboxes, dosettes, blister packaging provided by pharmacies, reminders or alarms, and automated pill dispensers [10]. Electronic medication adherence products are electronic products that are capable of dispensing medications in an attempt to address the limitations related to medication management capacity. These products have a multitude of features, including integrated alarms, multiple compartments, automatic components, security features, adherence report–generating features, and cloud connectivity [11]. However, there is limited research investigating the impact of electronic medication adherence products on medication adherence and, in particular, their use in older adults. In a previous qualitative study evaluating the views of patients and health care professionals with regard to electronic multicompartment medication devices, participants were asked to interact with 8 devices for 5 to 10 min using the think-aloud method to promote discussion in a focus group or in one-on-one interviews [12]. However, this study did not test whether participants were able to use the device for its intended purpose, for example, whether the participant could accurately fill the device, set the alarm, and access the medications from the device. Without testing these particular features, users may have difficulty fully establishing the usability and workload of a device. Therefore, we designed a mixed methods study to assess the usability, workload, and user experience of electronic medication adherence products for 3 stakeholder groups: older adults, caregivers, and health care providers. Caregivers and health care providers were included in conjunction with older adults to provide a holistic understanding of product use. Caregivers play a significant role in medication management of older adults and may at times be responsible for organizing medication administration aids, helping to administer medications, or many other medication management activities [13]. Similarly, health care providers often recommend medication adherence aids to patients experiencing nonadherence. The results of the usability and workload analysis from this mixed methods study have been published elsewhere [14].

### Objectives

The aim of this study is to explore the feedback and experience of stakeholders with regard to electronic medication adherence products, with particular emphasis on features, usefulness, and preferences.

## Methods

### Study Design

We conducted an exploratory qualitative study using semistructured interviews of stakeholders to assess the usefulness of, preference for, and features of electronic medication adherence products as part of a larger mixed methods study.

### Ethical Consideration

This study was reviewed by the Office of Research and Ethics of the University of Waterloo, and the study received ethics approval before recruitment and data collection. All participants were informed of the study, and they provided written consent before enrolling in the study.

### Study Participants

Purposive sampling techniques were used to recruit 3 types of stakeholders: older adults, caregivers, and health care providers. Individuals were eligible to take part in the study if they were (1) an adult aged 65 years or older who was taking one or more medications regularly and living independently, known as an *older adult participant*; (2) an adult who may or may not be living with the older adult participant and was involved in their medication management, known as a *caregiver*; or (3) health care providers (eg, physicians, pharmacists, nurses, and/or occupational therapists) who are involved in caring for the geriatric patient population in their practice and who may or may not have been involved in the direct care of an older adult participant. In addition, eligible participants were required to be able to speak English. Recruitment flyers and emails to organizations were used to recruit older adults and caregivers. In addition, a participant pool of older adults who indicated interest in participating in this study was approached via telephone. Health care providers were approached via email.

### Study Procedure

A total of 23 electronic medication adherence products were purchased; however, 1 was nonfunctional, and 1 was found to be nonelectronic but was still tested. Thus, 22 electronic medication adherence products were tested by participants (Table 1 provides a list of the electronic medication adherence products used). Products were chosen for purchase with the aim of representing different features, such as the number and type of compartments, audio and visual reminders, and medication tracking. Participants were invited to attend a 3-hour testing session at the School of Pharmacy Research Laboratory of the University of Waterloo. At the start of product testing, participants were introduced to a mock medication regimen containing placebo tablets, candy (Tic Tac), and placebo capsules and asked to familiarize themselves with the regimen by reading the labels of the medications. The mock medication regimen was designed by a pharmacist researcher and 2 research assistants after reviewing the literature on the most commonly dispensed medications for older adults and by examining the average number of medications taken by this population. Each participant tested 5 randomly selected products sequentially. At the start of each product test, participants were provided with instruction manuals made by the manufacturer. If the manufacturer’s instructions were not enclosed with the product, researchers found and printed web-based instructions for the participant. The testing tasks varied for each product but included steps such as opening and filling the medication tray, setting up the alarm, locking the device, and accessing the placebo pills from the device. Participants were given as much time as they needed to address all testing tasks and were not provided assistance unless requested.

**Table 1:** Electronic medication adherence products used in this study.

Name	Number of compartments^a^	Locking feature^b^	Portability^c^	Price, US $
GMS^d^ Med-e-lert automatic pill dispenser	28	Yes	No	70-109
LiveFine automatic pill dispenser and reminder	28	Yes	No	70-109
MedReady 1700 automated medication dispenser	28	Yes	No	≥109
MedSmart med-reminder and dispensing system	29	Yes	Yes	≥109
e-pill MedTime Station automatic pill dispenser with tipper	28	Yes	No	≥109
TimerCap travel size	1^e^	No	Yes	<30
TimerCap universal size	1^e^	No	Yes	<30
Jones medication adherence system 14-unit card	14	No	Yes	N/A^f^
Reizen vibrating pill box	5	No	Yes	<30
VitaCarry advanced pill case	7	No	Yes	30-69
Nishiki round pill box with alarm	7	No	Yes	<30
MedGlider system 1 with talking reminder	4	No	Yes	30-69
Patterson medical tabtime super 8	8	No	Yes	30-69
100-Hour pill reminder	3	No	Yes	<30
MedQ smart pill box	14	No	Yes	70-109
e-pill MedGlider home medication management system	7	No	Yes	30-69
MedCentre system	30	No	Yes	30-69
eNNOVEA Weekly Planner With Advanced Auto Reminder	14	No	Yes	70-109
e-pill Multi-alarm pocket XL	7	No	Yes	30-69
6 Grid pill storage case with alarm	6	No	Yes	<30
Itzbeen pocket doctor	0	No	Yes	<30
e-pill Weekly Accutab pill box organizer system^g^	21	No	No	30-69

^a^The total number of compartments a product contains to store medications.

^b^If the product has the ability to lock users from accessing their medications.

^c^If the product can be carried outside of the house using a purse or small bag.

^d^GMS: Group Medical Supply.

^e^Device has one compartment that can be accessed multiple times.

^f^N/A: not applicable.

^g^Device was found to be nonelectronic.

Following this testing phase, participants were invited to an optional one-on-one semistructured interview. The interview questions were developed by the research team and finalized through agreement. The research team consisted of 3 pharmacists and 1 occupational therapist who works closely with the geriatric patient population, 1 PhD student with extensive experience as a practicing pharmacist, 1 research assistant with 2 years of research experience, and 1 co-operative education student. All team members were female. Interview questions were developed to elicit information and feedback related to the experience of participants using the products (Textbox 1 provides a complete interview guide).

**Interview guide.**

Question 1

- What was one feature of any of the product that you liked the most? Why did you like this feature?
- What was one feature of any of the product that you did not like or liked the least? Why did you not like this feature?

Question 2

- What feature on any product did you find was the most useful? Why did you find this the most useful?
- What feature on any product did you find was the least useful? Why did you find this the least useful?

Question 3

- Do you use a medication administration aid currently? What do you use, and why do you use it?

Question 4

- Which of the products that you tested would you use for your own medication regimen? Why or why not?
- OR, would you recommend any of these products to your patients or parents to use for their medication regimen? Why or why not?

Question 5

- If you use an aid to help with your own medications, please compare this with the ones you tested today. Which do you prefer and why?

### Data Collection

A total of 2 research team members (AM and JI) conducted the interviews. All interviews took place between August 2018 and December 2018. If an older adult–caregiver dyad participated in the study, each person was interviewed separately. Each interview lasted for 15 to 30 min. All interviews were audio recorded using a Sony IC recorder ICD-PX470 and transcribed verbatim by 1 of 2 research team members (JI or AM), after which the accuracy of the transcription was established by the other research member. The interviews were transcribed using Microsoft Office Word.

### Data Analysis

The 6-phase framework by Braun et al [15] was used to perform thematic analysis [16]. The first interview transcript was individually coded by 2 research team members (JI and SF), after which preliminary codes were identified and compared with ensure coding consistency. The next 13 interviews were independently coded by the 2 researchers. A codebook was created to finalize the list of codes, and it contained the code name, code description, and quotes from the interview data. Percentage agreement and Cohen kappa were calculated between coders to ensure consistency in the first 13 interviews and was found to be 79% and 0.79, respectively. Any disagreement was discussed between the 2 researchers and resolved. The plan to bring any unresolved disagreements to a third researcher (TP) was not required. The remaining interviews were then analyzed by a single researcher (SF).

## Results

### Participant Demographics

A total of 39 participants were recruited for the larger study, of which 2 (5%) participants refused to participate in the optional one-on-one interview. Participants were not asked for a reason as to why they chose not to participate in the interview. Of the 37 participants, 21 (57%) were older adults, 5 (14%) were caregivers, and 11 (30%) were health care providers. There were 3 older adult–caregiver dyads. These dyads did not test the same products; they tested products independently and were interviewed individually (Table 2 shows the study participant characteristics).

**Table 2:** Study participant characteristics (N=37).

Participant characteristics			Values
**Older adults (n=21)**			
	**Gender, n (%)**		
		Male	10 (48)
		Female	11 (52)
	**Age (years)**		
		Mean (SD)	75 (6.5)
		Range	65-87
	**Total number of medications taken per participant**		
		Mean (SD)	7.7 (3.3)
		Range	1-13
	**Number of medications taken per participant by their type, n (%)**		
		≥5 Rx^a^, supplement, OTC^b^, and herbal	17 (81)
		≥5 Rx	10 (48)
		≥5 Supplement, OTC, and herbal	6 (29)
	**Medication schedule, n (%)**		
		Once daily	4 (19)
		More than once daily	17 (81)
	**Medication aids use, n (%)**		
		Yes	15 (71)
		No	6 (29)
	**Medication aids used in combination, n (%)**		
		Yes	4 (19)
**Caregivers (n=5)**			
	**Gender reported by the caregiver for their patient, n (%)**		
		Male	4 (80)
		Female	1 (20)
	**Age reported by the caregiver for their patient (years)**		
		Mean (SD)	73 (4.49)
		Range	69-79
	**Caregiver’s relationship with the patient, n (%)**		
		Family member	5 (100)
		Friend	0 (0)
		Other	0 (0)
**Health care providers (n=11)**			
	**Gender, n (%)**		
		Male	2 (18)
		Female	9 (82)
	**Occupation, n (%)**		
		Pharmacist	8 (73)
		Pharmacy student	1 (9)
		Occupational therapist	2 (18)
	**Years of practice**		
		Mean (SD)	8.8 (10.5)
		Range	0^c^-37
	**Number of older adults worked with in a typical week, n (%)**		
		<10	1 (9)
		10-20	4 (36)
		20-30	1 (9)
		>30	5 (45)
	**Medication aids recommendation, n (%)**		
		Yes	11 (100)

^a^Rx: prescription medications.

^b^OTC: over the counter.

^c^One health care provider was a pharmacy student and, thus, had 0 years of practice as a registered pharmacist.

**Figure 1:**
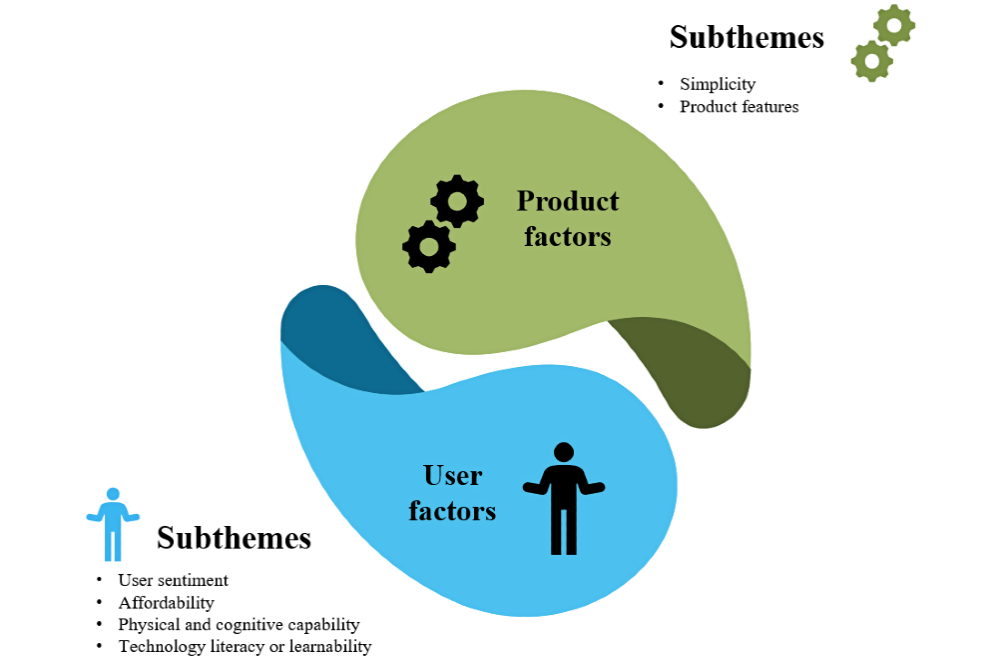
Themes and subthemes related to user experience with electronic adherence products.

A total of 39 codes were identified after the analysis. Codes were grouped into sub-themes, from which 2 themes and 6 sub-themes emerged (Figure 1 shows the themes and subthemes).

### Theme 1: Product Factors

*Product factors* was 1 of the 2 themes that emerged from our interview analysis. Product factors are factors associated with a product that can impact its use. The theme product factors were further divided into 2 subthemes: simplicity and product features.

#### Simplicity

Some electronic medication adherence products required multiple steps to set up, and although detailed information was provided by the manufacturer, it was not always perceived to be helpful by stakeholders. Stakeholders found some products easy to set up and simple to use, whereas they found other products to be complex. In general, the initial impression of complexity or simplicity impacted the overall impression and projected use of the product. Stakeholders did not appreciate products that required repetitive reviews of instructions to set up. One participant expressed their concerns through the following quote:

[This product is] just fiddley and difficult. I had to go over the instructions several times to understand that you had to lock, it wasn’t simple. And people, older people especially if they have a... long term illness, [they] want things that are simple. They don't want things that are going to be fiddley or [a] pain... [or] annoying to operate.

Older adult 003

#### Product Features

Product features are the characteristics, components, or capabilities of an electronic medication adherence product. This subtheme included the availability and usability of alarms, portability, restricted access to medication, and storage capacity.

##### Availability and Usability of Alarms

Stakeholders enjoyed electronic medication adherence products with audio or visual reminders, as they helped improve adherence. One participant described the importance of having the alarm as follows:

But no alarm, and that’s the downside to this [electronic medication adherence product]. That there’s no alarm on it. So, often I forget to take my Levodopa that I should be taking.

Older adult 004

However, the initial setup required for the alarm was considered to be an integral factor when choosing a product. Some products had small buttons or complex instructions that were not appealing to stakeholders.

##### Portability

In general, study participants commented negatively on products that were not portable, stating that products were too large, too noticeable, or visible in their home environment. Having a portable electronic medication adherence product was described as an important feature to consider when choosing a product for medication management, as a nonportable product may lead to missing a dose:

Like I’m going out for lunch with my women’s group. I can’t take this flying saucer with me. It’s not going to fit in my...the purse that I want to bring. So, I’m gonna miss... I’m probably gonna miss the dose by being out.

Older adult 027

##### Restricted Access to Medication

Another subtheme that emerged was the ability of a product to provide restricted access to medications, in turn allowing for secure medication administration and improved safety, that is, decrease the risk of inadvertently taking the wrong medication at the wrong time or double dosing. The ability to dispense the right dose at the right time was mentioned and preferred by most of our participants, as demonstrated by the following quote:

I liked that you couldn’t open it if you, like you...it’s impossible to take a second dose at the wrong time. It’s the only one of them that makes it so that you can’t essentially overdose on something.

Older adult 028

##### Storage Capacity

In this study, older adult participants took an average of 8 medications per day (range 1-13). Therefore, it is unsurprising that one feature frequently mentioned by participants when considering an electronic medication adherence product was its storage capacity. Storage capacity includes the number of the compartments and the number of days a product can store medications for. One caregiver discussed how none of the 5 products they tested during the study were suitable for the medication routine of their care recipient:

I couldn't use any of them for [my husband] because nothing [allows for] pills 8 times during the day. [For] the pillbox that we do use, I had to buy two sets. I have two different sets at home [to] accommodate all the pills [and] different times.

Caregiver 008

Health care providers similarly viewed a product’s storage capacity as important. One health care provider was quite pleased with how accommodating a product was with regard to its flexibility in compartments. This particular product had 28 compartments and allowed for 6 daily alarms:

Most of the patients who are senior, they are usually on 3-6 medications. So, and on multiple dosing, so 2 times a day, 3 times a day, 4 times a day. So, this [product] when you are [done with the] set up, it’s very well laid out for them.

Pharmacist 023

Overall, participants preferred electronic medication adherence products that had the ability to store multiple medications for several days or weeks. Users often found this feature very convenient and preferable when choosing a medication management aid.

### Theme 2: User Factors

User factors are defined as factors or abilities that can impact how a user uses the product effectively. User factors comprised 4 subthemes, including sentiment, affordability, physical and cognitive capability, and technology literacy or learnability.

#### Sentiment

Our interview analysis identified that participants felt various sentiments when using electronic medication adherence products. Sentiments can be defined as “an attitude, thought or judgment prompted by feeling” [17]. Some participants felt a sense of safety or peace of mind, whereas others were worried about the privacy of their medication intake process. A few participants also felt frustrated because the product was very complicated to set up and use, which impacted their overall impression of the product. These feelings were grouped under the following subthemes: sense of assurance, privacy, and frustration.

##### Sense of Assurance

An important aspect mentioned by participants when choosing an electronic medication adherence product was having a sense of assurance, so users did not have to worry about missing their medication dose. This assurance also provided participants with a sense of safety. For instance, one participant stated:

You don’t have to worry about “did I take my meds.”

Older adult 001

Health care providers shared the same feeling about having peace of mind when recommending electronic medication adherence products for their patients. One health care provider stated:

[The product] lets you know when you last took [the dose]... Even though you have an alarm telling you to take it... some people might forget that they actually took it and then might go to take... or check to see if there is another dose. So, this helps to know that it’s only been two or four or six hours since they last took it.

Pharmacist 006

This feeling of assurance was also associated with familiarity of a certain feature within an electronic medication adherence product. One participant preferred products that were similar to the pillbox they used at home and felt very comfortable using that particular product:

I like the pillbox [product name] because it’s huge and I'm used to doing that.

Older adult 002

##### Privacy

During this study, we tested products that ranged from small pillboxes to large automated dispensers. Most of our study participants disliked the idea of using large products and preferred products that were less noticeable. Participants found that large products brought attention to their medication regimen, a process that they would like to keep private. An example of a quote expressing concern about privacy is given below:

It was unobtrusive it wouldn't take up a lot of space to put it on the counter and nobody would really notice it. So, I like that about it.

Older adult 003

##### User Frustration

Another subtheme that emerged during our interview analysis was the feeling of frustration, particularly with the setup of the device. Participants did not prefer products that left them feeling frustrated. An older adult expressed their feelings when comparing a few electronic medication adherence products in terms of the complexity of the setup process:

This one [product] was fine too. I found it a little frustrating, the mode and the hour getting it set in holding it. A little bit frustrating. The container holding the pills was there it was clear and easy to see. And I liked that. Setting it was a little bit of annoyance.

Older adult 003

One of the health care providers also shared similar thoughts:

It was just difficult [and] confusing to set. And also, it's a nightmare for the pharmacist. You'd have to have perfect intervals which is annoying. And that [electronic medication adherence product] then requires whoever's using it to take a dose to then set the right interval. What if, you know, they set 12-hour intervals, but they don't want to wake up at 6am. It could be very annoying.

Pharmacist 023

Overall, the study participants preferred electronic medication adherence products that provide them with a sense of assurance and privacy and those that do not make them frustrated.

#### Affordability

Another subtheme that was identified during the interview analysis was the affordability of a product, although this area was not something that was probed for as part of our semistructured interview guide. Many of the older adults mentioned being on a fixed income and, as such, expressed interest and concern regarding the cost of products. Health care providers expressed similar concerns, as shown in the following quote:

Cost may be a big issue because majority of my patients are under government coverage and asking them to pay a dollar is very, very difficult. And if you ask them to pay for something, unless it’s sponsored by the government or something, I don’t foresee us getting a patient to buy this. They’ll say they can take their medication in which we know they can’t. But still they will not pay to buy this.

Pharmacist 020

#### Physical and Cognitive Capability

Many participants mentioned that the physical and cognitive ability to use electronic medication adherence products was a crucial aspect when considering the use of a product. The use of electronic medication adherence products can be impacted by a user’s ability to access the medication from the device without any difficulties. Some products required manual dexterity to operate, including accessing medications from the device and setting the alarm. For example, individuals are required to press small buttons, flip switches, or rotate portions of the device to operate the product optimally. Similarly, good visual or hearing abilities are required to respond to audio-visual reminders. Some of these products also necessitate the significant cognitive capacity among users to understand how to operate and respond to alarms and prompts. These concerns were shared across all stakeholder groups. One older adult expressed the following concerns:

Some of the buttons were very hard to manipulate. They hurt your fingers or they're too small.

Older adult 014

Health care providers were also concerned about their patients experiencing difficulty using electronic medication adherence products, especially if they had an impairment or medical condition that could affect their manual dexterity. As one of the health care providers quoted:

The little one. It’s just so hard. It’s not hard for me to open it, but it’s small. It [would be] hard for an older adult that has some peripheral neuropathy or arthritis or something that would make it difficult or not useful to use this.

Occupational therapist 038

#### Technology Literacy and Learnability

Stakeholders felt that they needed to acquire new information related to technology to use some products adequately. This was problematic for some stakeholders because of the nonfamiliarity with technology and the technological complexity of the product. One health care provider expressed their concern through the following quote:

I do like the fact that there is a phone reminder, but then the person using it has to be tech-savvy as well. I don't know...I mean there are more older adults now who are able to use smart phones but the majority of my patients over the age of 80, they can barely use any smart device.

Pharmacist 020

## Discussion

### Principal Findings

This study highlights how the factors of an electronic medication adherence product along with the user factors impact the preference of a stakeholder to choose a product for medication management. For daily use of a product, users must be able to learn how to set up and regularly use the product appropriately. If the process of learning how to use the product is complex, it may impact the usability of the product, leading to improper product use. Usability can be defined as the manner in which a user interacts with a product and the ability of the user to perform the required tasks to use the product [18]. In many cases, electronic medication adherence products are advertised by manufacturers as simple to use and generally do not indicate whether the help of a health care provider or a caregiver is required. The usability study conducted as part of this larger mixed methods study showed that the usability and workload of electronic medication adherence products vary greatly among stakeholders and highlighted the need to assess these factors to provide guidance to health care providers regarding product recommendations to their patients [14].

Technology plays a significant role in every aspect of life [19,20]. Electronic medication adherence products are innovative products that may require some familiarity with technology. Research has shown that the interaction of older adults with technology is not similar to the interaction of younger adults and children with technology because of age-related cognitive and physical changes [21]. Individuals who are facing challenges in these areas, lack familiarity with technology, or feel uncomfortable relying on technology may dislike or be anxious about adopting a technology-based solution [21]. This uneasiness was expressed by older adults who participated in this study. However, with the continued use of a product, the technical aspects may become less challenging and this uneasiness may decrease.

In addition to product usability, this study showed that stakeholders prefer electronic medication adherence products that accommodate complex medication regimens, incorporate alarms, are secure and portable, require minimal technology use, and are affordable. Older adults with multiple comorbidities often take complex therapy regimens on a regular basis [8]. Managing multiple medications at variable times during the day is a complex task and often leads to confusion and improper medication use [8,12]. Many older adults in this study noted how some products would not accommodate their daily medication regimen and, therefore, indicated that the storage capacity of a device would be an important factor when choosing an electronic medication adherence product.

In addition, patients may forget to take their medications or take them inappropriately because of age-related cognitive impairment [22,23]. To address this factor, numerous interventions have been developed, including alarms and audio-visual reminders [24]. These reminders include auditory sounds, vibrations, and/or flashing lights. Studies have reported that electronic medication packaging devices with audio-visual alarms or reminder systems can positively impact medication adherence [23,25]. According to this study, although an alarm or reminder function was considered to be important when choosing an electronic medication adherence product, the effort required to set up the alarm should also be considered. Therefore, to integrate electronic medication adherence products into daily use, electronic medication adherence products with reminder or alarm functions should be user-friendly, simple to set up, and easy to operate.

Studies have also shown that users prefer devices that are small in size and those that do not attract attention when used in public [24,25]. Previous studies have indicated that older adults prefer medication management products that are portable and do not interfere with their ability to leave the house [8]. Similar results were shown by our study participants, as they preferred electronic medication adherence products that could be easily carried or were less disruptive when leaving the home as they could bring their medications with them during day trips for outings with family and friends.

In general, older adults live on fixed or low incomes and, thus, may have difficulties affording an electronic medication adherence product. Some electronic medication adherence products have lower costs, whereas others range from a few hundred to thousand dollars. Although these products are being manufactured and marketed for improving medication adherence, the lack of reimbursement by provincial or private health care insurance plans prevents older adults from using these products. Studies have shown that people who are unable to afford costs for their medications are more likely to be nonadherent to long-term therapies for chronic medical conditions such as asthma, diabetes, heart failure, and depression [26,27]. Similarly, individuals may be reluctant to pay out of their pockets for a medication adherence aid that they may not be able to afford. Therefore, the cost of the electronic medication adherence product is another important feature to consider when choosing these products.

It is likely that there is no one product that will meet the needs of every older adult. Future iterations of and developments in electronic medication adherence products should consider the characteristics of patients when designing products. For example, the features of an electronic medication adherence product required to meet the needs of a 75-year-old patient with a history of arthritis, hypertension, and stroke who has limited vision and loss of function and is on a complex medication regimen with multiple drugs are considerably different from the needs of a highly functioning 65-year-old patient who is forgetful about once daily dosing of their antihypertensive medication. Caregivers may prefer a product that provides restricted access to medications so that their care recipient is not at risk of medication overdose, whereas another caregiver may want a device that automatically dispenses the medication and has a loud audio-visual alarm for their care recipient. Similarly, a 60-year-old person with Parkinson disease who is taking medications 6 times per day will find the storage capacity of an electronic medication adherence product to be a far more important factor when it comes to selecting an electronic medication adherence product. An individualized approach should be used to select a particular electronic medication adherence product, depending on the needs of the patient, to gain its full benefit for medication management. Manufacturers developing these products should collaborate with patient partners to address the needs of the population.

### Strengths and Limitations

A major strength of this study was the involvement of multiple stakeholders in testing and providing feedback on electronic medication adherence products. Another strength was the inclusion of a wide range of electronic medication adherence products, from simple alarm-based pillboxes to highly sophisticated automated dispensers. As participants were interviewed after testing 5 products, rather than after each product, participants had the option of comparing all 5 electronic medication adherence products in their interviews. As a result, a limitation of this study was that participant responses may have been driven by their experience with 1 or 2 products, rather than discussing all products tested. To combat this, researchers ensured all products were visible during the interview to allow for better recall and prompted for additional details if no product was mentioned during the interview. Another limitation of this study was that interview data from stakeholder groups were not analyzed separately and, therefore, do not provide a detailed report of each group’s feedback for product preference.

### Conclusions

In conclusion, this study demonstrated that preference for a particular electronic medication adherence product depends on multiple factors, including, but not limited to, the storage capacity, security, cost, and size of the device. Just one electronic medication adherence product will not be suitable for everyone. Therefore, health care providers should consider patient-related factors such as cognitive and functional capability to operate a device, medication regimens, and product features to choose the right product for the right patient. The manufacturers of these electronic medication adherence products should also consider the involvement of users in the beginning stages of product development for these technologies to ensure high acceptability, user friendliness, and affordability for end users. Policy makers should consider subsidizing the cost of electronic medication adherence products to make them affordable for people who are chronically ill and are on long-term therapies, allowing for a reduction in costs related to nonadherence in the health care system.
